# Network Pharmacology-Based Approaches of *Rheum undulatum* Linne and *Glycyrriza uralensis* Fischer Imply Their Regulation of Liver Failure with Hepatic Encephalopathy in Mice

**DOI:** 10.3390/biom10030437

**Published:** 2020-03-12

**Authors:** Su Youn Baek, Eun Hye Lee, Tae Woo Oh, Hyun Ju Do, Kwang-Youn Kim, Kwang-Il Park, Young Woo Kim

**Affiliations:** 1 Institute for Phylogenomics and Evolution, Kyungpook National University, Daegu 41566, Korea; rhodeus@nate.com; 2 School of Medical Science, Kyungpook National University, Daegu 41566, Korea; eun90hye@nate.com; 3 Korea Institute of Oriental Medicine, Daegu 41062, Korea; taewoo2080@kiom.re.kr (T.W.O.); dododo@kiom.re.kr (H.J.D.); lokyve@kiom.re.kr (K.-Y.K.); 4 College of Veterinary Medicine, Gyeongsang National University, Jinju 52828, Korea; kipark@gnu.ac.kr; 5 School of Korean Medicine, Dongguk University, Gyeongju 38066, Korea

**Keywords:** *Rheum undulatum*, *Glycyrriza uralensis*, hepatic encephalopathy, MMP-9, neuroinflammation

## Abstract

*Rheum undulatum* and *Glycyrrhiza uralensis* have been used as supplementary ingredients in various herbal medicines. They have been reported to have anti-inflammatory and antioxidant effects and, therefore, have potential in the treatment and prevention of various liver diseases. Considering that hepatic encephalopathy (HE) is often associated with chronic liver failure, we investigated whether an *R. undulatum* and *G. uralensis* extract mixture (RG) could reduce HE. We applied systems-based pharmacological tools to identify the active ingredients in RG and the pharmacological targets of RG by examining mechanism-of-action profiles. A CCl_4_-induced HE mouse model was used to investigate the therapeutic mechanisms of RG on HE. We successfully identified seven bioactive ingredients in RG with 40 potential targets. Based on an integrated target–disease network, RG was predicted to be effective in treating neurological diseases. In animal models, RG consistently relieved HE symptoms by protecting blood–brain barrier permeability via downregulation of matrix metalloproteinase-9 (MMP-9) and upregulation of claudin-5. In addition, RG inhibited mRNA expression levels of both interleukin (IL)-1β and transforming growth factor (TGF)-β1. Based on our results, RG is expected to function various biochemical processes involving neuroinflammation, suggesting that RG may be considered a therapeutic agent for treating not only chronic liver disease but also HE.

## 1. Introduction

Hepatic encephalopathy (HE) is a decline in neuropsychiatric function observed in patients with acute or chronic liver diseases [1,2]. The clinical symptoms are so diverse that it may appear as a subtle impairment in mental state, but it can lead to coma [1]. There are numerous explanations of why liver dysfunction can lead to encephalopathy; however, the most common clinical mechanism through which HE develops is an increase in the level of blood ammonia, which could be removed by sodium benzoate [1,2,3,4]. In a healthy body, the nitrogen-containing compounds that remain following the food digestion process are transported through the portal vein to the liver, where most such compounds are metabolized. If the liver cells or the metabolism processes are impaired, nitrogen wastes containing a large amount of ammonia can accumulate in systemic circulation. Small nitrogen-containing molecules, such as ammonia, can pass through the blood–brain barrier (BBB), and astrocytes in the cerebral cortex can absorb ammonia to convert glutamate into glutamine [3,5]. An excessively high glutamine level in astrocytes may increase osmotic pressure and the activity of the inhibitory GABA system, leading to a shortage in the energy supply to the brain [3,5]. 

Commonly utilized pharmacologic HE treatments tend to focus on lowering the blood ammonia level [3,4]. Clinically, two classes of medications—non-absorbed disaccharides, including lactulose and lactitol; and nonabsorbable antibiotics, including neomycin and rifaximin—are normally used [2,3,4]. However, both these two classes are known to show negative side-effects, such as diarrhea, electrolyte disturbances and resistances, which can limit long-term effectiveness and patient compliance [4,6]. Therefore, it is necessary to be simultaneously considered for effective treatment, including the presence of oxidative stress or neurotoxins, as well as changes in neurotransmission, GABA-ergic pathways and energy metabolism. During such consideration, it is wise to consider the potential of herbal medicine treatments, which have been widely applied in a variety of medical fields due to their high efficiency and stability [6,7].

Rhubarb (*Rheum undulatum*) is a perennial plant belonging to the Polygonaceae family, and the components of dried roots of *R. undulatum* are reported to have therapeutic efficacy in oriental traditional medicine against various diseases [8,9]. Herbal preparations of *R. undulatum* are used as a stimulant laxative with excellent efficacy in reducing inflammation in the liver, large intestine, and kidney. Previous studies have reported that its components have potent antitumor, antithrombotic, and antioxidant properties [10,11,12]. A review argued that rhubarb, alone or combined with other herbs, can be used to treat HE and to improve treatment efficacy, which was promising but not based on solid empirical evidence [8]. Ning Z. et al. (2017) reported that the rhubarb-based Chinese herbal formulae (RCHF) might be effective at treating hepatic encephalopathy using meta-analysis in China. Their data showed that the rhubarb-based Chinese herbal formulae (RCHF) patients had significant improvements in their ammonia and alanine aminotransferase levels in their blood when compared to the other groups of patients that received other treatments [13]. Moreover, a few studies demonstrated that rhubarb contained multiple biological activities probably associated with BBB function, including anti-oxidation, anti-inflammation, and inhibition of aquaporin expression [14,15]. However, no research has yet been reported on which molecular mechanisms rhubarb treats HE.

A previous our study showed a significant protective effect of an *R. undulatum* and *Glycyrrhiza uralensis* extract mixture (hereafter RG) against hepatic oxidative injury in two model systems: hepatocytes treated with arachidonic acid (AA) plus iron and mice with CCl_4_-induced liver injury [16]. We found the best ratio of RG (1:10) in vitro and applied at mice model of acute hepatitis [16]. *Glycyrrhiza glabra* Fisch have been used to treat a wide array of illnesses in herbal and traditional medicines for thousands of years [16,17]. Triterpene saponins, such as glycyrrhizin, uralsaponin, liquorice saponins, and glycyrrhetinic acid; as well as flavonoids, such as liquiritin, liquiritigenin, Isoliquiritin, isoliquiritigenin, and isolicoflavonol, are reported to be responsible for the pharmacological properties of licorice [17]. In the present study, we analyzed RG to elucidate the mechanism-of-action of each component, to identify the components that are thought to have a key role and, ultimately, to predict the therapeutic effects of these components by constructing a drug-target-disease network model. We performed in vivo and in vitro experiments to determine whether RG has an effect on HE, and if so, by what molecular mechanisms. Our results are expected to provide theoretical and in vivo empirical evidence that RG may be used as an effective drug in the treatment of HE.

## 2. Materials and Methods

### 2.1. Reagents

Anti-claudin 5 was obtained from Santa Cruz Biotechnology (Santa Cruz, CA, USA). Horseradish peroxidase conjugated goat anti-rabbit and goat anti-mouse IgGs were purchased from Novus Biological (Centennial, CO, USA). Trizol was obtained from Invitrogen (Invitrogen, Carlsbad, CA, USA) and reverse transcriptional polymerase chain reaction (RT-PCR) kit was obtained from Promega (Promega, Madison WI, USA). Sennoside A, emodin, chrysophanol, aloe-emodin, rhein, glycyrrhizin acid, liquiritigenin, isoliquiritigenin, and other reagents were purchased from Sigma-Aldrich (St. Louis, MO, USA). The reagents for ultra-performance liquid chromatography (UPLC) analysis were methanol (Junsei for the high-performance liquid chromatography (HPLC), acetonitrile (JT BAKER for the HPLC), and then Water (Tertiary distilled water).

### 2.2. Preparation of RG Extracts

*Rheum undulatum* (200 g) and *Glycyrriza uralensis* (200 g), which are warranted as a standard herb for medicine by Korea Food and Drug Administration, were purchased from Sejong pharmacy (Daegu, South Korea) and were boiled in 1500 mL of distilled water for 3 h. This aqueous mixture was passed through a 0.2 mm filter (Nalgene, New York, NY, USA). The filtrate was concentrated under reduced pressure at 50 °C and lyophilized into powder. The extraction yields of *R. undulatum* and *Glycyrriza uralensis* were 11.33% and 15.61%, respectively.

### 2.3. Profiling the Chemical Contents of RUE and GUE by Ultra-Performance Liquid Chromatography

**Figure 1:**
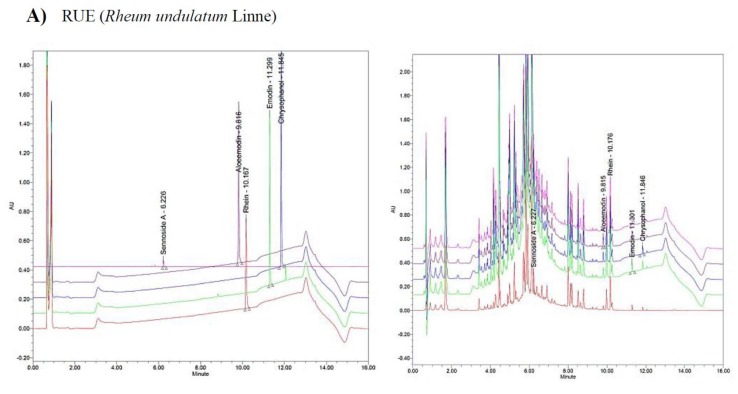

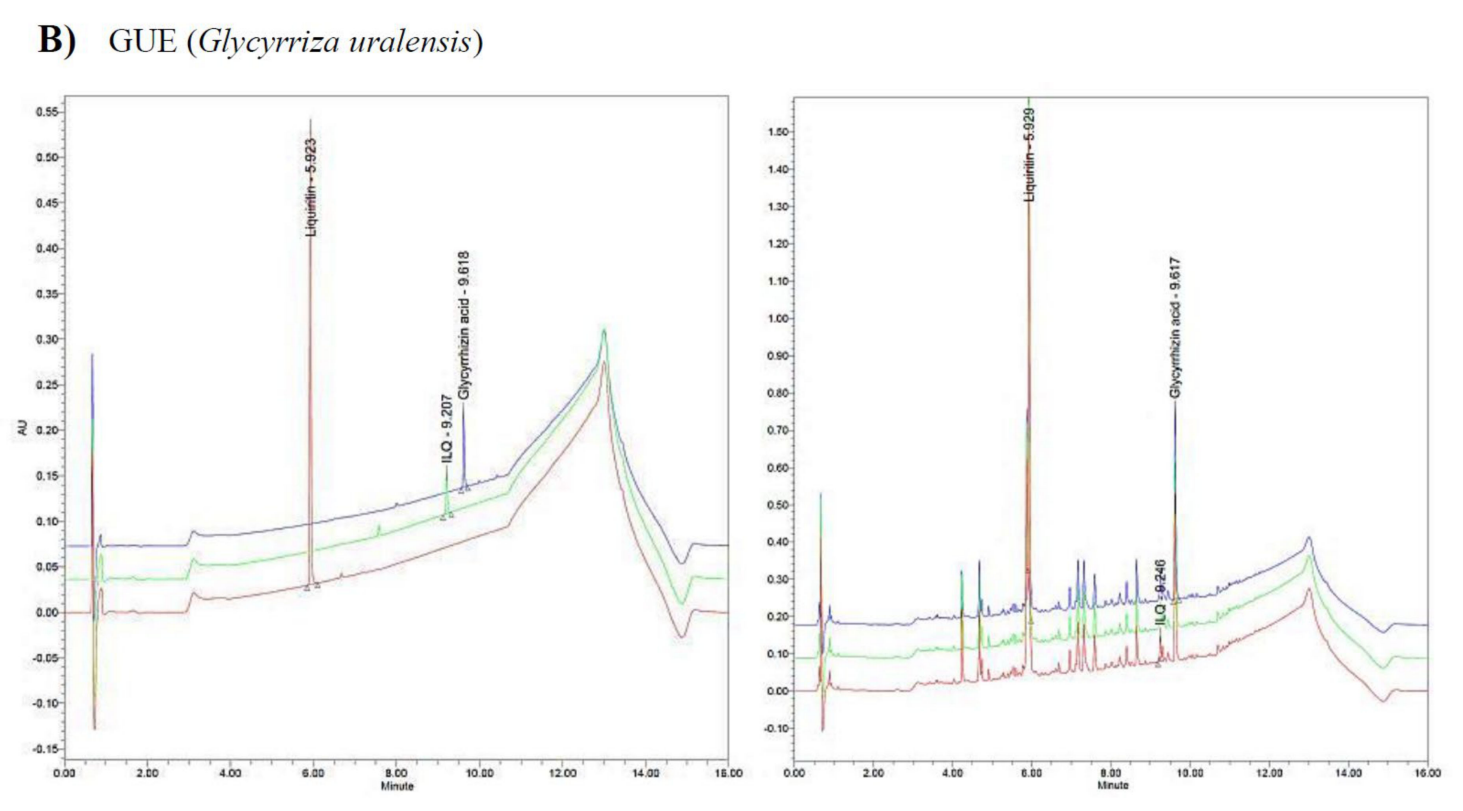
Characterization of RUE (*Rheum undulatum* Linne) (**A**) and GUE (*Glycyrriza uralensis*) (**B**) by UPLC chromatogram. (**A**) UPLC chromatogram of five commercial standards (left) and marker compounds in GUE (right). Each peak represents sennoside A (340 nm), emodin (254 nm), chrysophanol (254 nm), aloe-emodin (254 nm), and rhein (254 nm), respectively. (**B**) UPLC chromatogram of theww commercial standards (left) and marker compounds in RUE (right). Each peak represents glycyrrhizin acid (254 nm), liquiritigenin (280 nm), and isoliquiritigenin (280 nm), respectively.

Ultra-performance liquid chromatography) analysis was performed using an ACQUITY ultraperformance LC system equipped with ACQUITY photodiode array (PDA) detector (Waters Corporation, Milford, CT, USA) based on the methods described in a previous study [18]. ACQUITY BEH C18 column (Waters Corporation; 1.7 μm, 2.1 × 100 mm) was used to elute the compounds off and Empower software was applied to tract and compare the peak data. The standard compounds were melted by methanol and DMSO. Next, they were prepared based on a standard undiluted solution containing 1 mg/mL. The PDA analysis wavelengths were sennoside A (340 nm), emodin (254 nm), chrysophanol (254 nm), aloe-emodin (254 nm), rhein (254 nm), glycyrrhizin acid (254 nm), liquiritigenin (280 nm), and isoliquiritigenin (280 nm), respectively. Samples were 2 mL, and the flow rate was 0.4 mL/min. Five and three potential ingredients were collected from *R. undulatum* and *G. uralensis*, respectively (Figure 1 and Table 1).

### 2.4. Systems Pharmacology-Based Analysis

Molecules with oral bioavailability (≥30%) and drug-likeness indices (≥0.18) were considered to select the target compounds that could be used for our study [19,20]. Oral bioavailability (OB), the capable of being delivered to systemic circulation after oral administration, is one of the most important pharmacokinetic parameters in drug screening [20]. The drug-likeness index was used to examine the herbal ingredients under consideration whether they are suitable as medicaments by quantifying the structural similarities to all drugs registered in the DrugBank database (http://www.drugbank.ca/) [19]. The potential targets and diseases of the candidate compounds were predicted by Traditional Chinese Medicine Systems Pharmacology Database and Analysis Platform (TCMSP) (http://lsp.nwsuaf.edu.cn/) [21]. The protein targets predicted were examined for their functional annotation based on the sequence similarities to the proteins registered in UniProt (http://www.uniprot.org/). Finally, the potential targets were imported to the DAVID (http://david.abcc.ncifcrf.gov) for gene ontology (GO) enrichment analysis. The GO terms with *p*-values of less than 0.05 were filtered out, and functional annotation clustering analysis was implemented for the remaining terms to identify the pharmacological and biological processes. The likelihood networks connecting candidate compounds, potential targets and their related diseases were constructed by Cytoscape 3.2.1 (Bethesda, MD, USA). The topological properties of the networks generated were analyzed using the Plugins (Network Analysis, Analyze Network) of this software.

### 2.5. Animals

Balb/c male mice (6 weeks old) purchased from Charles River Orient Bio (Seongnam, Korea) were maintained under standard condition (23 ± 1 °C; 50 ± 5% humidity; 12-h light/dark cycle). All animal experimental procedures were approved by the Institutional Animal Care and Use Committee of Daegu Haany University and were conducted in accordance with the guidelines of the National Institutes of Health (Protocol # DHU2016-047). The mice were allocated randomly into three treatment groups: ‘vehicle-treated control’ (*n* = 8), ‘CCl_4_’ (*n* = 8), and ‘CCl_4_ + RG’ (*n* = 8). Mice were orally administered by either RG (10 mg/kg of RUE plus 100 mg/kg of GUE, dissolved in water; for CCl_4_ + RG) or water (for vehicle-treated control and CCl_4_) three times per week for 4 weeks. To induce liver damage in groups CCl_4_ and CCl_4_ + RG, CCl_4_ (0.5 mL/kg of body weight and 1:9 diluted in corn oil) was intraperitoneally injected into mice twice a week for 4 weeks. All the mice were sacrificed on day 28 using carbon dioxide chamber.

### 2.6. Behavioral Tests

An open-field test was designed to evaluate our animals’ basal activity and its change across time in response to our pharmacological treatments in accordance with the procedure described in a previous study [22]. Video tracking with recording was conducted under dim lighting condition to examine the movement pattern within the peripheral and central zones in an open field for 30 min. Movement patterns were quantified based on the distance traveled and time spent in each zone using a video tracking system (SMART V3.0; Panlab, Barcelona, Spain).

### 2.7. Brain Immunohistochemistry

The brain sections from three groups of mice (vehicle-treated control, CCl_4_ and CCl_4_ + RG) were fixed in 10% neutral buffered formalin, then embedded in paraffin by tissue processor and embedding center. Tissue blocks were sectioned in 4 μm thick ribbons and applied on slide glass. Two slides were randomly selected for each group, and hematoxylin and eosin (H&E) staining was performed on selected slides according to the standard protocol. After deparaffinization in xylene, tissue sections were hydrated in EtOH and stained in hematoxylin for 5 min. After bluing, tissue sections were stained with eosin for 10 s. Followed by eosin, tissue sections were dehydrated and cleared before mounting.

These slides were used for immunohistochemical detection of glial fibrillary acidic protein (GFAP), which was carried out according to the protocol described below. A BenchMark XT autostainer (Ventana Medical Systems, Tucson, AZ, USA) was used in immunohistochemistry (IHC). Coated glass slides were loaded with 4-μm-thick liver tissue sections and heated at 60 °C for 2 h. EZ prep. solution (Ventana Medical Systems) was used to deparaffinize followed by cell conditioning 1 solution. Cell conditioning 1 solution (Ventana Medical Systems) was used as heat-induced antigen retrieval agent. Anti-GFAP antibody (Abcam, Cambridge, UK) was diluted to 1:100 and applied on slides for 60 min. Universal DAB detection kit (Ventana Medical Systems) was used to detect specific reaction and nucleus was stained with hematoxylin followed by bluing reagent.

### 2.8. Blood Analysis

Collected whole blood was incubated at room temperature for 2 h and centrifuged for 5 min at 5000 rpm to isolate plasma. Plasma ALT was analyzed by using an automated blood analyzer (Fuji Dri-Chem NX500i, Fuji Medical System Co., Ltd., Tokyo, Japan).

### 2.9. Liver Histopathology

The largest lobe of each liver removed was fixed in 10% neutral buffered formalin, then embedded in paraffin by tissue processor and embedding center. Tissue block was sectioned in 4-μm-thick ribbon and applied on slide glass. After deparaffinization in xylene, tissue sections were hydrated in EtOH and stained with either H&E or with Masson’s trichrome for collagen fibers. Following staining, tissue sections were dehydrated and cleared before mounting. The histological changes were observed under a light microscope (Nikon, Tokyo, Japan).

### 2.10. Western Blot Analysis

Western blot analysis was performed as previously described [18]. Briefly, the brain tissues were lysed with radioimmunoprecipitation assay (RIPA) buffer (Thermo Scientific, Rockford, IL, USA) at 4 °C and each protein sample (30–50 μg) was separated by 10% sodium dodecyl sulfate–polyacrylamide gel electrophoresis (SDS–PAGE) and transferred onto polyvinylidene fluoride (PVDF) membranes (Millipore, Bedford, MA, USA). The protein bands were detected with WesternBright ECL (Amersham Biosciences, Piscataway, NJ, USA) and a gel-doc image analyzer (Vilber Lourmat, France). The band intensity was quantified using Image J 1.42 software (NIH; Bethesda, MD, USA).

### 2.11. Real-Time RT-qPCR Analysis

Total RNA was extracted from the brain tissues using Trizol (Invitrogen, Carlsbad, CA, USA) following the manufacturer’s instructions [18], and primer sets were shown in Table 2. The mRNA levels of matrix metalloproteinase-9 (MMP-9), transforming growth factor beta 1 (TGF-β1), and interleukin 1β (IL-1β) were compared by calculating the crossing point (C_p_) value and were normalized by glyceraldehyde-3-phosphate dehydrogenase (GAPDH) using LightCycler 96 relative quantification software (Roche, München, Germany). A melting curve analysis was performed following the amplification to verify the accuracy of the amplicons.

### 2.12. Statistics

Mean ± standard deviation (SD) was provided for all quantitative data. All comparisons were examined based on either one-way ANOVA followed by Bonferroni’s post hoc test or a two-tailed Student’s *t*-test. The criterion for statistical significance was set at *p* < 0.05, but an indication was added when *p* was less than 0.05, 0.01, or 0.001.

## 3. Results

### 3.1. Systems Pharmacology-Based Approach

Based on our ultra-performance liquid chromatography (UPLC) results, five (sennoside A, emodin, aloe-emodin, chrysophanol, and rhein) and three (glycyrrhizin acid, liquiritigenin, and isoliquiritigenin) representative components were identified from the *R. undulatum* and *G. uralensis* extracts, respectively (Figure 1 and Table 1). We compared between two peaks of the commercial standards and of corresponding herbal component. All but sennoside A were considered target compounds to be tested in this study because they exceeded our oral bioavailability (OB) and drug-likeness evaluation criteria. Sennoside A was excluded as it had a very low OB value (less than 0.1). In addition, given that the absorption rate is very low in the small intestine when a component enters via the oral cavity and can be easily converted to rhein anthrone by various enzymes associated with intestinal microorganisms [23,24], it was not necessary to consider sennoside A as a target component.

**Figure 2:**
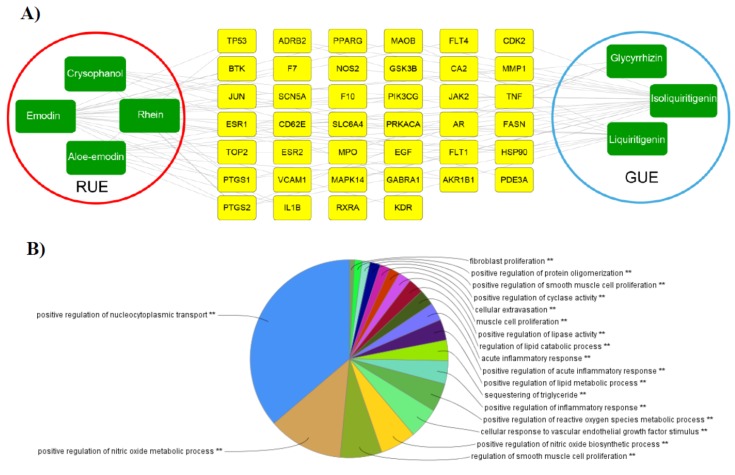

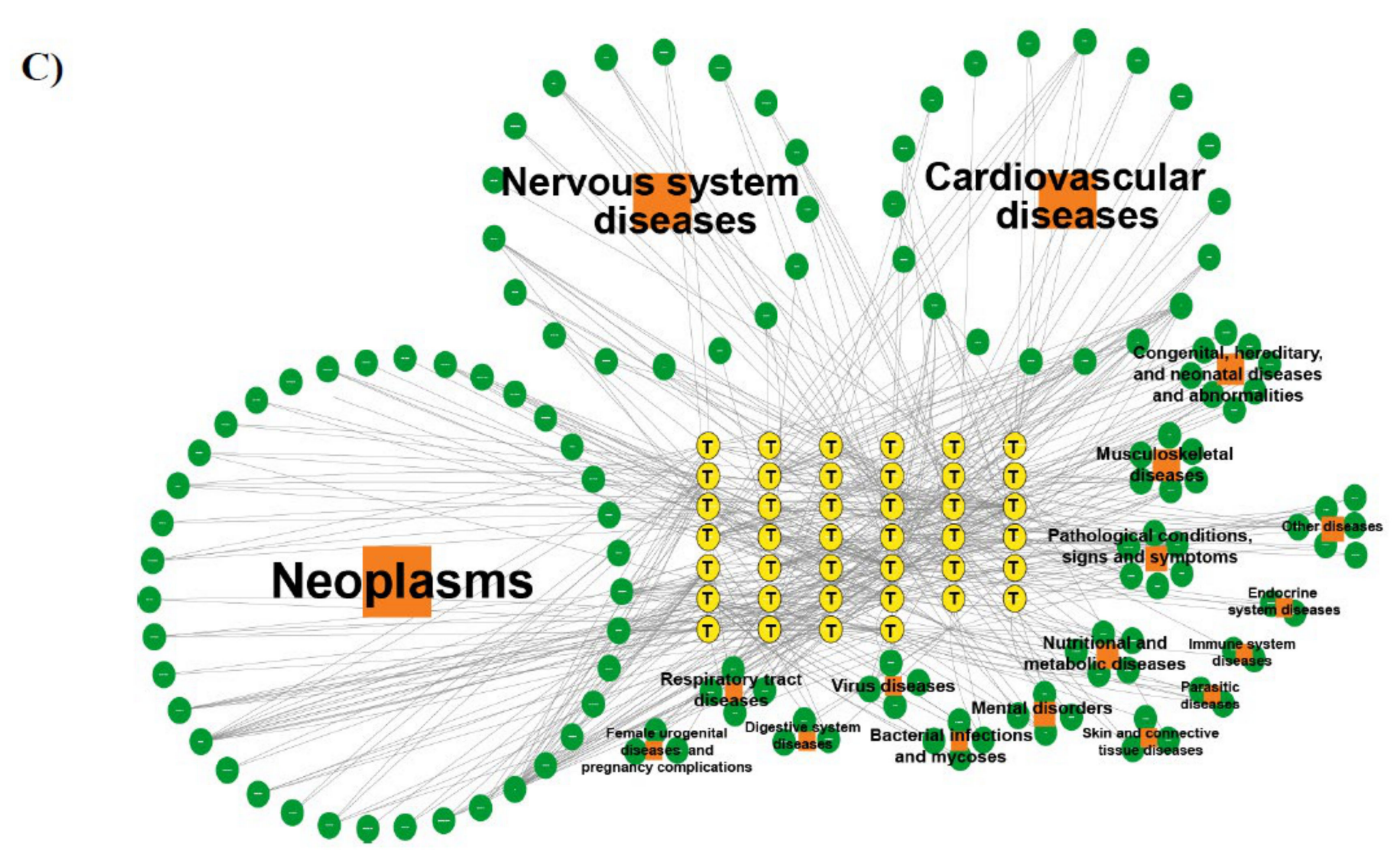
Systems pharmacology-based approach. (**A**) Compound–target network (C–T network). The C–T network was constructed by connecting the candidate compounds (green rectangles) of *R. undulatum* (red circle) and *G. uralensis* (blue circle) with their potential targets (yellow rectangles). The C–T network was composed of 141 compound–target links generated from the connection of the 7 candidate compounds to 40 targets. (**B**) ClueGO analysis of the predicted targets. The pie chart represents the molecular function, immune system processes and reactome pathways of the targets identified in the network analysis. (**C**) Target–disease network (T–D network). In the T–D network, candidate targets were connected to the related diseases. Target proteins (40, yellow ellipses) were connected to 135 diseases (green circles), which could be assigned into 18 separated groups (orange squares).

To understand the mechanism associated with the RG components’ HE-related medicinal action at a systems level, a compound–target network was constructed to uncover the underlying interactions (Figure 2). The compound–target network with colour-coded nodes to indicate components (green) and candidate targets (yellow) comprised 141 interactions connecting the 7 selected RG components to 40 targets (Figure 2A). Two components, emodin (degree = 34) and isoliquiritigenin (degree = 30), appeared to exhibit the most versatile functions, with many of their functions associated with the pharmacological regulation of HE (Figure 2A). As the network indicates, the RG components and their targets are intertwined; thus, it is better to interpret a component as having a complex function rather than having a specific function. In an effort to illuminate the therapeutic mechanism of the 40 selected targets, we applied ClueGO, a Cytoscape (https://cytoscape.org/) plugin, to perform a biologically relevant interpretation of the potential targets. The majority of targets were associated with oxidoreductase, nucleocytoplasmic transport and inflammatory response (Figure 2B).

All 40 of the targets were entered into pharmacology database platforms including DrugBank, Therapeutic Target, and PharmGkb to obtain a list of potentially related diseases. A total of 135 diseases, belonging to 18 groups suggested by the MeSH Browser (2017 MeSH), were identified. The target–disease interactions were visualized by using a network format (Figure 2C). Of the 135 diseases identified, 38 were found to be related to neoplasms, 17 to nervous system diseases, and 18 to cardiovascular diseases. These three disease groups are all reported to have the potential to be improved by the administration of RG.

### 3.2. RG Improves CCl_4_-Induced Behavioral Damage

**Figure 3:**
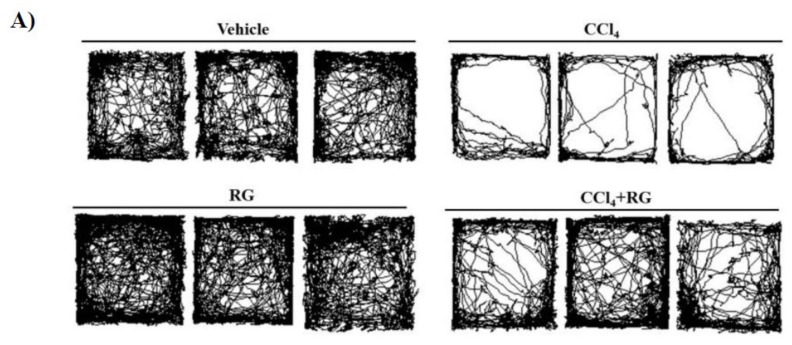

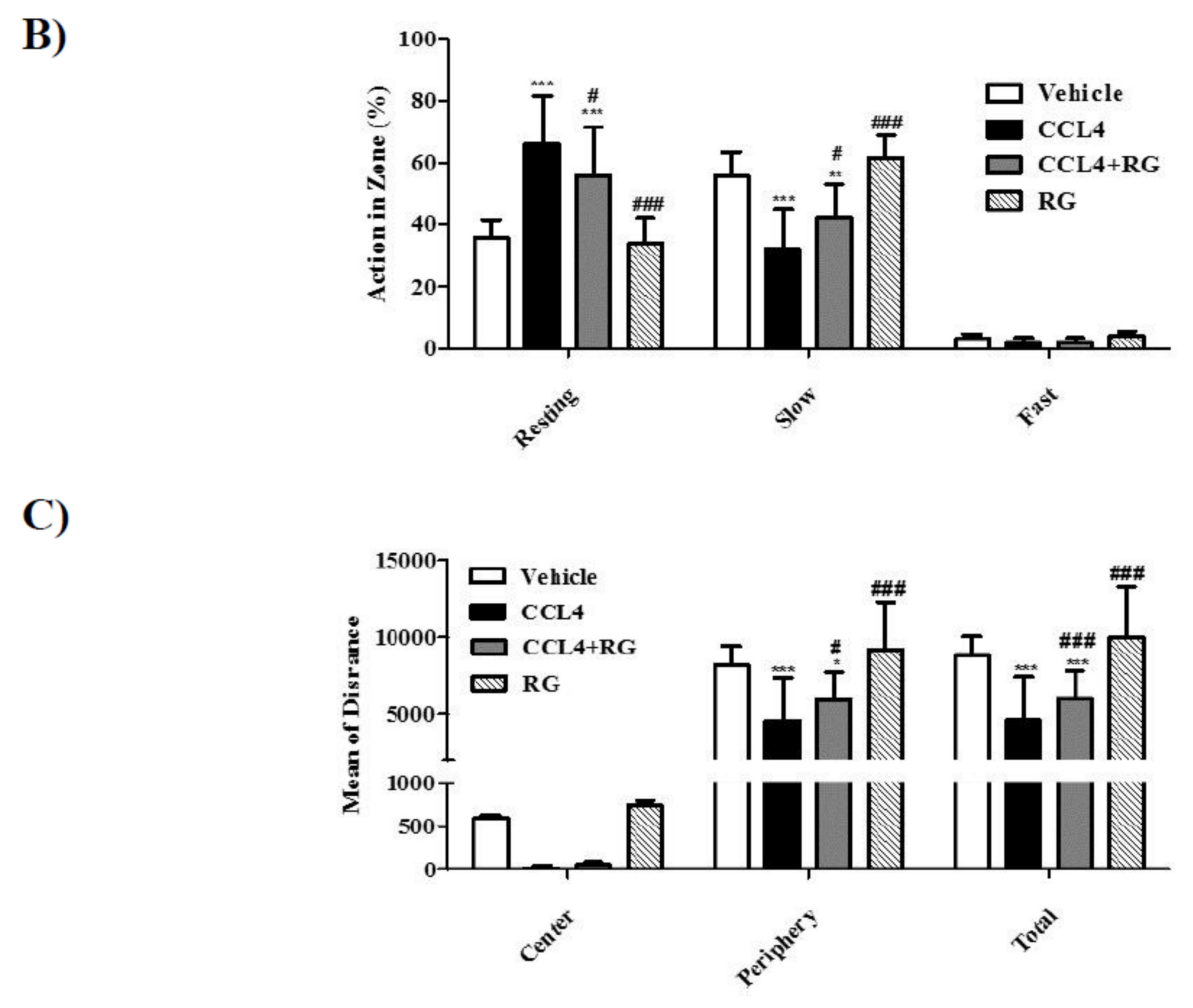
RG ameliorates neurobehavioral changes in CCl_4_-induced hepatic damage model. Neurobehavioral changes observed in the open field tests (OFTs) in CCl4-induced hepatic damage model. (**A**) Representative traces of mouse movement in the experimental field. (**B**) % action categories (resting, slow, and fast) in both center and periphery zones by mice. (**C**) Distance travelled in both center and periphery zones by mice. Data were analyzed for statistical significance using ANOVA with Tukey’s test for ad hoc multiple comparison implemented in GraphPad Prism. Data were presented as mean ± SD for three independent experiments. *p < 0.05, **p < 0.01, ***p < 0.001 when compared with vehicle group; ^#^*p* < 0.05, ^##^*p* < 0.01, ^###^*p* < 0.001 when compared with group CCl_4_.

Because it had been reported that RG protects against liver damage, we focused on nervous system related diseases among the three key disease groups. To determine the effects of hepatic damage on behavior, we performed open-field tests on mice. Based on the methods described in a previous study [25], locomotion activities were quantified by measuring distance travelled and time spent in the centre or periphery of the open field for 30 min. The results showed that exposure to CCl_4_ was associated with depressive-like symptoms (Figure 3A). Compared with the level of resting activities of the vehicle group, the group with CCl_4_-induced hepatic damage (CCl_4_ group) showed a significant increase in the amount of time spent resting (Figure 3B). In addition, the level of slow activities was decreased in the CCl_4_ group compared to the level in the vehicle group (Figure 3A,B). Furthermore, the CCl_4_ group travelled a shorter distance than the vehicle group (Figure 3A,C). In contrast, depression did not appear in the RG-treated group, and behavioral symptoms of that group were similar to those of the vehicle group that did nothing more than CCl_4_ group (Figure 3). Furthermore, the group treated with CCl_4_ plus RG (CCl_4_ + RG group) exhibited significantly more activity and greater distance travelled than those in the CCl_4_ group (Figure 3). Furthermore, in the RG treatment group, there was a greater distance travelled than that in the control group (Figure 3A).

### 3.3. RG Ameliorates CCl_4_-Induced Histopathological Changes of Brain

**Figure 4:**
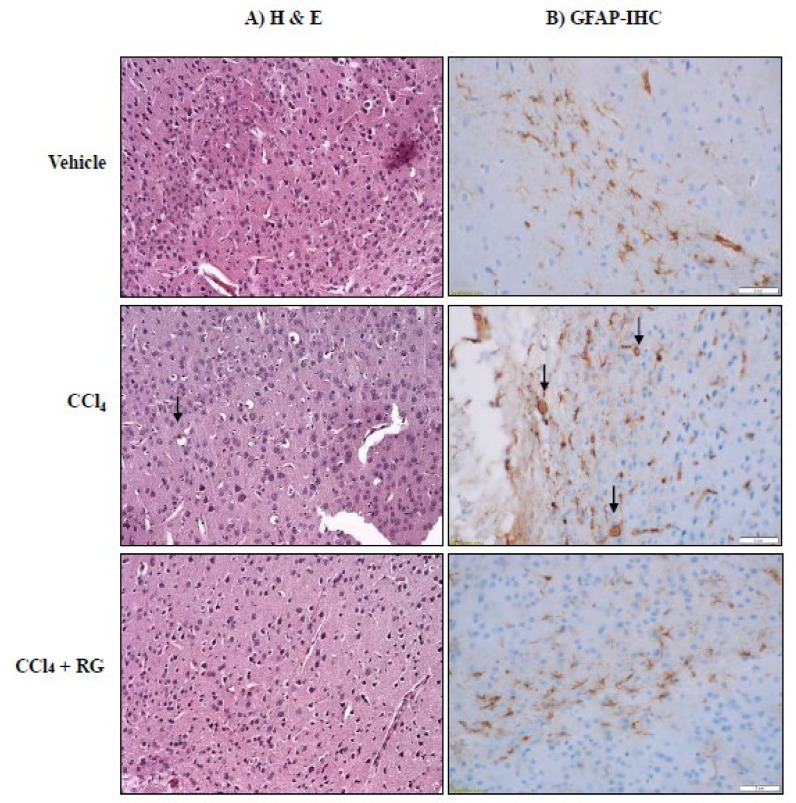
RG ameliorates CCl_4_-induced histological changes in cerebral cortex. Mice were given with repeated injections of CCl_4_ (5 mg/kg, 2 times/week, 4 weeks), whereas vehicle mice instead received saline (*n* = 4). The CCl_4_ + RG group was treated with RG (10 mg/kg of R plus 100 mg/kg of G) with CCl_4_. After four weeks of CCl_4_ injection, mice were sacrificed. (**A**) Histopathological change (when stained with hematoxylin and eosin; H&E) and (**B**) astrogliosis (glial fibrillary acidic protein, GFAP) in cerebral cortex. Magnification: 400×.

We investigated histopathological changes in the cerebral cortex by examining H&E-stained samples (Figure 4). The CCl_4_ treatment produced lesions in the cerebral cortex that exhibited swollen astrocytes with large vesicular nuclei and prominent nucleoli (Figure 4). However, the RG combination treatment (10 mg/kg of R plus 100 mg/kg of G) protected astrocytes in the cerebral cortex from exhibiting the lesional damages that are observed following CCl_4_ treatment (Figure 4A).

Astrogliosis was examined by quantifying GFAP-immunoreactive cells in the area of the cerebral cortex (Figure 4B). CCl_4_ seemed to induce intensive GFAP staining and to increase process complexity. However, the number of GFAP-positive cells in the CCl_4_ + RG group was reduced when compared to that in the CCl_4_ group (Figure 4B). Overall, it appears that CCl_4_ administration increases the number of activated astrocytes and RG treatment can significantly reduce that effect.

### 3.4. RG Inhibits the BBB Disruption and Neuroinflammation

**Figure 5:**
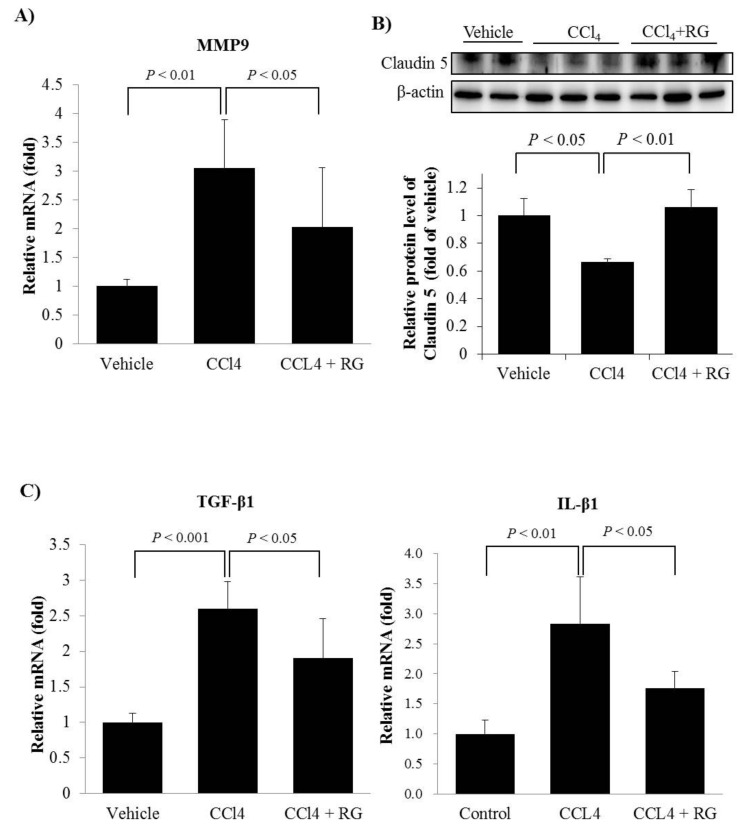
RG suppresses BBB disruption and inflammation in mice with CCl4-induced HE. Mice were treated with CCl_4_, and RG as described in Figure 3. RT-qPCR analysis of the mRNA expression levels of (**A**) MMP-9, (**C**) TGF-β1, and (**D**) IL-1β. (**B**) The protein levels of claudin-5 were assessed by western blot, in which β-actin served as a loading control. Protein levels were presented as relative band intensities to control (vehicle treated) group. Data were represented by mean ± SD.

The effect of RG on the CCl_4_-induced increase in brain endothelial cell permeability was determined by analysing MMP9 mRNA in mouse brain tissue samples. The mRNA expression of MMP9 was significantly upregulated in brain tissues of the CCl_4_ group, but that upregulation could be blocked by pre-treatment with RG (Figure 5A). To verify the effectiveness of RG in preventing BBB disruption, the expression of claudin-5, a tight junction protein, was assessed by undertaking an immunoblotting analysis. The level of claudin-5 was significantly decreased in the CCl_4_ group, compared to that in the control group (Figure 5B). However, the presence of RG led to a greater increase in the level of claudin-5 protein in the CCl_4_ + RG group than in the CCl_4_ group (Figure 5B), indicating that RG has a protective function in preventing BBB disruption.

Neuroinflammation is a fundamental immune response in the central nervous system (CNS) through which the brain reacts to diverse pathogens passing through the damaged BBB. Therefore, we examined the mRNA expression levels of the representative pro-inflammatory cytokines IL-1β and TGF-β1 in the cerebral cortex. Real-time RT-qPCR analysis showed that the presence of RG effectively inhibited the CCl_4_-induced expressions of IL-1β and TGF-β1 (Figure 5C).

### 3.5. RG Ameliorates CCl_4_-Induced Liver Injury 

**Figure 6:**
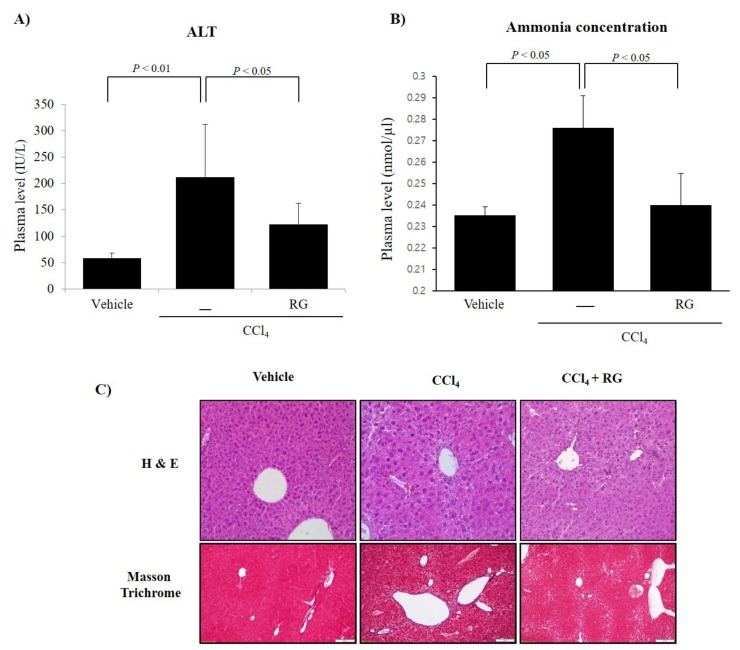
RG protects CCl_4_-induced liver toxicity in mice. Mice were given with repeated injections of CCl_4_ (5 mg/kg, 2 times/week, 4 weeks), whereas vehicle mice instead received saline (*n* = 4). The CCl_4_ + RG group was treated with RG (10 mg/kg of R plus 100 mg/kg of G) with CCl_4_. After four weeks of CCl_4_ injection, mice were sacrificed. (**A**) The activities ALT and (**B**) ammonia were assayed by using semi-automated blood chemistry analyzer after 4 weeks of treatment. Data were represented by the mean ± SD. (**C**) Representative photomicrographs of liver sections processed for H&E (upper row) and Masson’s trichrome (lower row) staining with vehicle, CCl_4_, and CCl_4_ + RG. Scale bars = 80 μm.

To examine the hepatic protective effects of RG, we established a chronic hepatic cirrhosis mouse model by using injections of CCl_4_. Repeated injections of CCl_4_ (5 mg/kg twice per week for 4 weeks) significantly elevated the serum ALT level, indicating that liver damage had occurred (Figure 6A). However, RG pre-treatment markedly inhibited the elevation of the serum ALT level caused by the CCl_4_ injections (Figure 6A). It is known that the increase of ammonia in serum is a representative indicator of hepatic encephalopathy as well as liver disease. Our results of reducing ammonia in serum increased by CCl_4_ with RG treatment provide further evidence that RG can mitigate live injury caused by CCl_4_ (Figure 6B).

In addition, we observed histopathological differences between groups in livers that had been stained with H&E and Masson’s trichrome (Figure 6B). CCl_4_ treatment can produce degenerative damages to liver tissue, typically, centrilobular necroses such as ballooning and vacuolation (deposition of lipid droplets) of hepatocytes and the infiltration of inflammatory cells (Figure 6C). However, pre-treatment with RG markedly minimized the CCl_4_-induced liver damage (Figure 6C). 

## 4. Discussion

There are many reports indicating that a mixture of various medicines can be used to obtain a better effect than that when the medicines are used separately. Based on a previous report, extracts from *R. undulatum* and *G. uralensis* were able to provide synergistic antioxidant and hepatic protective effects against AA and iron-induced oxidative stress as well as against CCl_4_-induced acute liver injury [16]. Considering that HE is a frequent disease associated with chronic liver failure [2], in this study, we investigated whether RG could directly reduce HE or its symptoms. The study consisted of several experimental and analytic steps.

First, we constructed a systems-based pharmacological model that integrated OB predictions, target predictions and interaction networks in order to provide insights into the effects and mechanisms of RG on HE. After identifying the components of RG and choosing among those components those that were deemed worth prescribing, we examined how these candidate ingredients interact with certain targets in human physiology. Based on our target–disease network results, it can be assumed that some of the components identified in RG could be used for the treatment of several diseases in addition to HE. This multi-target potential is a benefit of many herbal medicines, and it appears that RG is likely to treat HE in a manner that can interact with other important targets in the overall human physiology rather than concentrating its effects only on one disease. In addition, the discovery of a close relationship in the target–disease network with neoplasms and cardiovascular disease indicates another asset of RG as a therapeutic agent.

Second, we examined whether RG had sufficient efficacy to reduce symptoms in HE induced by CCl_4_ in mice. Administration of CCl_4_ to mice induces chronic liver failure, which can lead to CNS changes similar to those seen in HE. CCl_4_ is one of the most widely used chemicals that cause liver injury. It is activated in liver microsomes by several functional enzymes dependent on the cytochrome P450 family, and that activation generates an abundance of free radicals, which leads to lipid peroxidation within the cells. In addition, CCl_4_ can covalently bind to cellular lipids and proteins, which may lead to dissociation of cell membrane structure and increase membrane permeability [26]. CCl_4_ is often used to create rat HE models [27]. In a previous study, it was reported that rats treated with CCl_4_ or alcohol had very similar symptoms to those of HE [28]. It was also reported that BALB/c nude mice showed rapid and obvious liver injury and brain dysfunction following intraperitoneal injection of CCl_4_ [29]. As predicted, we observed that CCl_4_ administration resulted in a severely impaired neurological score, decreased activity, and diminished cognitive function. However, RG treatment recovered those effects to a considerable level, though not to the level in the control subjects. Likewise, astrocyte swelling in the brains of CCl_4_-treated mice was remarkably moderated by RG treatment. Such results show that RG has a protective effect on the brain against the development of HE.

The major ingredients of RG and their potential targets shown in our results indicate that RG could ameliorate disruption of the BBB by regulating the molecules involved in forming tight junctions, such as MMP and vascular cell adhesion molecule 1 (VCAM1). MMP-9 can digest major proteins in the capillary endothelial BBB and in tight junctions. MMP-9 is known to degrade occludin and claudin-5 in focal cerebral ischemia [30], and it has been reported that it affects the expression of occludin, claudin-5, and ZO-1 and ZO-2 levels in early diabetic retinopathy [27,31,32]. MMP-9 upregulation was previously reported to open the BBB during the later stages of HE [31]. During ammonia-induced HE, for example, the upregulation of MMP-9 disrupted the expression of major tight junction proteins, and when MMP-9 was pharmacologically inhibited, their expressions were restored to near control level [33]. In our results, an increased level of MMP-9 mRNA expression in the cerebral cortex was observed following CCl_4_ administration. However, RG treatment resulted in inhibition of MMP-9 gene expression. In addition, the inhibition of claudin-5 expression observed in the brains of CCl_4_-treated mice was completely moderated by RG treatment. These results affirm the results of previous studies that showed that rhubarb could maintain BBB integrity in traumatic brain injury treatment [14] or intracerebral haemorrhage [15]. Our data suggest that RG can effectively attenuate CCl_4_-induced BBB damage in mice, raising the possibility that RG or its active components may be considered as potential neuroprotective drugs useful in relieving the severe symptoms of HE.

If the BBB is breached and circulating neurotoxic agents and inflammatory mediators penetrate into the brain, astrocytes absorb them and, simultaneously, secrete a variety of pro-inflammatory cytokines including IL-1β, IL-6, and TNF-α [34]. Cytokines may directly induce astrocytes to diffuse into brain parenchyma and activate transcription factors in astrocytes in order to increase the production of cytokines. In a recent study, increased brain efflux of IL-1β, TNFα, and IL-6 was identified in patients with uncontrolled intracranial hypertension due to acute liver failure [35,36]. Intracerebral synthesis of cytokines may be a primary cause for the characteristic astrocyte swelling often seen in this kind of condition. In this study, we show that that CCl_4_ markedly increased the levels of IL-1β and TGF-β1 in mouse cerebral cortex, and that increase was effectively inhibited by the presence of RG.

Sometimes, the general consumers expect that the some hepatoprotective agents could reduce the severity of extra-hepatic manifestation of liver failure (e.g., hepatic encephalopathy). Like this, it is inferred that reduced encehphalopathy after RG could be off-label uses, not a direct effect of RG on brain mechanism. It might be partly explained by a reduction in severity of liver failure.

## 5. Conclusions

Our results demonstrate the therapeutic effects on chronic HE models of a mixture of extracts from *R. undulatum* and *G. uralensis*. We applied theoretical and computational approaches to determine the active ingredients of those extracts and to identify their pharmacological targets by exploring their mechanism-of-action profiles. In the animal models, RG consistently relieved the symptoms of HE by protecting BBB permeability via downregulation of MMP-9 and upregulation of claudin-5. The effects of RG are mediated by a variety of biochemical processes involving neuroinflammation. In this regard, the use of RG appears to be advantageous as it can provide more effective and rapid treatment effect than other medicines that are involved in only one or two processes. Thus, RG may be considered as a therapeutic agent for treating not only chronic liver disease but also HE.

## Figures and Tables

**Table 1:** Chemical formula and mass accuracy of potential ingredients.

Sample	Identity	Chemical Formula	Mass Accuracy (ppm)
RUE (*Rheum undulatum* Linne)	Sennoside A	C_42_H_38_O_20_	145.85 ± 7.080
	Emodin	C_15_H_10_O_5_	1.20 ± 0.012
	Chrysophanol	C_15_H_10_O_4_	0.064 ± 0.001
	Aloe-emodin	C_15_H_10_O_5_	2.11 ± 0.616
	Rhein	C_15_H_8_O_6_	29.47 ± 0.447
GUE (*Glycyrriza uralensis*)	Glycyrrhizin acid	C_42_H_62_O_16_	325.91 ± 6.8
	Liquiritigenin	C_15_H_12_O_4_	124.25 ± 3.7
	Isoliquiritigenin	C_15_H_12_O_4_	6.08 ± 0.7

**Table 2:** Primers used in real-time PCR analysis in this study.

Genes	Sense	Antisense
*MMP-9*	5′-TCCCTCTGAATAAAGTCGACA-3′	5′-AGGTGACAAGGTGGACCATG-3′
*IL-1β*	5′-CAGGATGAGGACATGAGC-3′	5′-CTCTGCAGACTCAAACTCCA-3′
*TGF- β1*	5′-GAGGTTTGCTGGGGTGAG-3′	5′-CAGCACGAGGAGGAGCAG-3′
*GAPDH*	5′-AACGACCCCTTCATTGAC-3′	5′-TCCACGACATACTCAGCAC-3′

## Abbreviations

ALT	alanine aminotransferase
BBB	blood–brain barrier
CCl4	carbon tetrachloride
C-T network	compound–target network
H&E	harri’s hematoxylin and eosin
HPLC	High-performance liquid chromatography
GABA	γ-Aminobutyric acid
IHC	immunohistochemistry
IL-1β	Interleukin 1 beta
MMP-9	matrix metallopeptidase 9
OB	Oral bioavailability
TGF-β1	Transforming growth factor beta 1
UPLC	ultra-performance liquid chromatography
